# Real-time teleteaching in medical physics

**DOI:** 10.2349/biij.4.1.e13

**Published:** 2008-01-01

**Authors:** M Woo, KH Ng

**Affiliations:** 1 Department of Medical Physics, Odette Cancer Centre, Toronto, Canada; 2 Department of Biomedical Imaging, Faculty of Medicine, University of Malaya, Kuala Lumpur, Malaysia

**Keywords:** Remote real-time teaching, teleteaching, distant education, medical physics

## Abstract

Medical physics is a relatively small professional community, usually with a scarcity of expertise that could greatly benefit students entering the field. However, the reach of the profession can span great geographical distances, making the training of students a difficult task. In addition to the requirement of training new students, the evolving field of medical physics, with its many emerging advanced techniques and technologies, could benefit greatly from ongoing continuing education as well as consultation with experts.

Many continuing education courses and workshops are constantly being offered, including many web-based study courses and virtual libraries. However, one mode of education and communication that has not been widely used is the real-time interactive process. Video-based conferencing systems do exist, but these usually require a substantial amount of effort and cost to set up.

The authors have been working on promoting the ever-expanding capability of the Internet to facilitate the education of medical physics to students entering the field. A pilot project has been carried out for six years and reported previously. The project is a collaboration between the Department of Medical Physics at the Toronto Odette Cancer Centre in Canada and the Department of Biomedical Imaging at the University of Malaya in Malaysia. Since 2001, medical physics graduate students at the University of Malaya have been taught by lecturers from Toronto every year, using the Internet as the main tool of communication.

The pilot study explored the different methods that can be used to provide real-time interactive remote education, and delivered traditional classroom lectures as well as hands-on workshops.

Another similar project was started in 2007 to offer real-time teaching to a class of medical physics students at Wuhan University in Hubei, China. There are new challenges as well as new opportunities associated with this project. By building an inventory of tools and experiences, the intent is to broaden the real-time teleteaching method to serve a wide community so that future students entering the field can have efficient access to high-quality education that will benefit the profession in the long term.

## INTRODUCTION

The medical physics profession is well-positioned to benefit from remote education. The profession is relatively small; most of them are concentrated in the USA, UK and Western Europe, as well as in other parts of the world such as Japan, Australia, Korea, and other countries. However, there is a growing demand for medical physicists, mainly because of the complex nature of medical care in a world of advanced technology. There is a projected shortage of well-trained physicists, as predicted by the American Association of Physicists in Medicine (AAPM) [1] in its requirement for accreditation of graduate student and residency programs [2].

The demand is probably even more significant in many rapidly developing countries, where the field of medical physics has not hitherto existed, at least not in a well-defined and structured way. In order to meet this growing demand cost-effectively, local students are often recruited into new training programs and curricula in existing universities and hospitals. This has created a great need for knowledgeable instructors in the field of medical physics.

Such experts do exist, but they are usually concentrated in areas or countries where the field of medical physics is well-established. In order to facilitate the dissemination of knowledge, the AAPM has established different committees with the objective of international exchange and education in the profession [3].

Parallel to the growing demand and technological advances in the medical physics field, an even bigger explosion has been occurring in the field of global communication, primarily in the form of the Internet and universal video/audio communication. Much of the educational and training demand has helped create the field of distant education, not just in medical physics, but in all disciplines of education.

## OVERVIEW OF DISTANT EDUCATION

### Asynchronous vs Synchronous

Within the field of distant education, different modes of delivery have developed. One classification of the delivery methods is asynchronous versus synchronous. The distinction is that the former refers to off-line teaching using educational modules while the latter refers to real-time on-line teaching. In other words, the former simply means that students are accessing pre-assembled modules of educational material which generally take on many different formats such as virtual libraries, online journals, and specialised software such as WebCT, Blackboard [4], etc., which enable educators to prepare course modules effectively and in a well-organised manner. There are several good reviews of the different asynchronous modes of educational tools, dealing with general questions associated with distance learning [5, 6, 7].

In contrast, synchronous education very closely resembles the traditional classroom environment where the course organiser or lecturer interacts directly with the student. The interaction may take on different forms depending on the technological tools involved, but the main feature is that the instructor and the student, or groups of them, are not physically in the same room. In other words, there could be multiple instructors and students at multiple locations, interacting directly at the same time, as if they were 'virtually' located in the same room.

The merits of asynchronous versus synchronous modes have been investigated extensively [8]. In summary, asynchronous delivery provides flexibility for both teachers and students, and is very cost-effective especially when delivered on a large scale. The tools of asynchronous delivery are well-established and most universities now offer course modules based on this.

### Synchronous Mode of Distant Education

Synchronous delivery provides the opportunity for more individualised interaction, which often is most valuable when difficult concepts are involved and when each individual student might have different issues of difficulty. It is in this area that expert guidance is invaluable and irreplaceable. The nature of synchronous delivery, however, renders the process relatively expensive, and perhaps because of that, the tools for synchronous delivery are not as well-developed and established. However, this is rapidly changing and the traditional high-end products used by corporate businesses for video-conferencing are gradually becoming accessible for comparatively small-scale educational projects. In fact, many commercial education projects have been spruced up, such as remote home tutoring and online language education. Personal audio / video communication over the Internet has become a common practice in our society.

Asynchronous delivery has become a key tool in mainstream university education. Interestingly, even though most universities have adequate facilities for synchronous delivery, it is still rare to find such applications in large institutions. So it may be fair to say that synchronous delivery in educational settings is still in its infancy.

Some of the common platforms for synchronous delivery have been comprehensively summarised [9]. These tools will be discussed in more detail in Sections III and IV below.

## DISTANT EDUCATION IN MEDICAL PHYSICS

There are many examples to show how the asynchronous mode has been applied in the field of medical physics. The Emerald project was one of the earliest contributions which promoted the role of teleteaching and the Internet in general in medical physics education [10]. The AAPM has a well-established virtual library [11], while the Biomedical Imaging and Intervention Journal (BIIJ) website [12] contains recorded sessions of talks at conferences and symposia. Most medical physics journals are now available online. There are also various forms of online tools, such as the Standford Dosimetry Tool [13], as well as the MedPhys Wiki tool being developed by the AAPM Task Group TG-131. In addition, a lot of distant education material for medical physics are available on the websites of many major institutions and organisations in the profession, such as the International Atomic Energy Agenecy (IAEA) [14], Radiological Society of North America (RSNA) [15] and American College of Radiology (ACR) [16] .

On the other hand, the synchronous mode has not been well-exploited. There are a few reported applications [17], and the AAPM has a Webex service for committee meetings, where committee members meet online and communicate directly via a common PC screen or via regular telephone conference calls. However, there is only a peripheral element of education in this application.

Direct broadcast of seminars are also gradually taking place. One of the authors' host hospitals, Sunnybrook Health Science Centre, conducts live webcasts of many medical physics seminars, as well as radiation oncology rounds [18]. However, this service is still in its infancy and the talks usually end up being recorded and archived to become an asynchronous educational medium. This is not because of the limitations of technology, but because of the profession’s unfamiliarity with the format and the concept of interacting by posting live questions remotely to the speaker.

For the field of medical physics, however, the synchronous mode has definite advantages over the asynchronous mode. Firstly, there is the advantage of being able to delve into difficult concepts interactively, rather than conveying a large amount of information without opportunity for feedback. Problem-solving situations, which often come up in medical physics, are most effectively dealt with in interactive sessions, where students' misinterpretation of some key concepts can be much more readily identified.

Another very useful aspect of synchronous delivery is in the area of 'hands-on' procedures. This can be divided into two categories where the synchronous mode would be very valuable. Firstly, many medical physics tools involve software programs such as treatment planning systems, dosimetry analysis systems (such as film analysers, beam scan analysers, etc), and image analyser software. A valuable part of the medical physics training program is to share a PC desktop and demonstrate how a treatment planning system works and also why various parameters (such as IMRT cost functions, CTV margins, DVH analysis, etc) are selected.

Secondly, one could even go beyond sharing the PC desktop screen, to demonstrating various physics procedures to students in a remote location. It is feasible to use a portable video camera to go through a Linac QA process or a TG-51 calibration in a remote session. The instructor explains each step and the students can question any complicated details, or ask the instructor to zoom in on a piece of the equipment.

One often reads about 'amazing' stories featuring remote procedures, such as remote robotically-operated surgery. Instead of the huge cost required for the equipment in such highly critical procedures, the above-mentioned educational application does not require any sophisticated or specialised equipment, or any dedicated network connection. Therefore, the technological hurdles or associated costs are minor deterrents to these 'ambitious' projects.

As mentioned before, a medical physicist's expertise is often rare in areas where it is most needed. And in many aspects, because of the fast-paced development in the field, it can be difficult to find experts even in well-established communities. Developing synchronous delivery into a routine role would address this issue, as it would allow valuable resources to be deployed effectively and optimally.

The experts in the field would also benefit greatly from the synchronous mode. They can address the training of individual groups efficiently and often quite flexibly. A training session can now include simply the actual class time rather than all the travelling overhead. Short sessions can easily be arranged, and there is the flexibility of conducting the session away from the office and outside of office hours. As mentioned before, the delivery can be expanded to instructors and students at multiple sites, to offer even better efficiency and flexibility.

## PROJECTS ON SYNCHRONOUS (REAL-TIME) TELETEACHING IN MEDICAL PHYSICS

In view of the demand for distant education in medical physics and with the technological tools in place for exploiting synchronous teleteaching, the authors launched a pilot project in 2001 to explore the feasibility of, and various aspects associated with, the concept. The project has been reported [19], and is summarised again below. More information can be obtained from the RRTL (Remote Real-Time Teaching and Learning) website [20].

### Pilot project between University of Toronto and University of Malaya

A collaboration was set up in 2001 between the Department of Medical Physics at the Odette Cancer Centre (then known as the Toronto-Sunnybrook Regional Cancer Centre, and affiliated with the University of Toronto) in Toronto, Canada and the Department of Biomedical Imaging at the University of Malaya in Kuala Lumpur, Malaysia.

A class of medical physics graduate students from the University of Malaya attended lectures provided by lecturers in Toronto, using the Internet as the main tool of communication. There were two aspects to the project. The first was to experiment with the method of real-time interactive communication provided, as well as the logistics of the communication, such as the time differences and the availability of facilities. The second aspect was to determine the optimal contents and formats of such a remote education project.

The main ‘hardware’ tools used included a personal computer (PC) at both the instructors’ and students’ ends, with both systems connected to the Internet, a regular telephone connection with a speakerphone, as well as other personal computer accessories, such as a web-camera, a microphone and speaker set, a drawing tablet, etc. The software tools included a screen-sharing program, GoToMyPC [21], by which the class could view the instructor’s computer screen and the lectures that were provided using, for example, Power Point slides. The screen-sharing program also allowed the class to interact directly by typing or drawing on a whiteboard, as well as by directly controlling the mouse of the instructor’s computer to interact with specific programs, with the instructor’s permission. Another software program, Skype [22], provided the audio/video communication between the two sides. An alternative to that was to use the regular telephone for audio communication only. As part of the study, many of these tools were tested to determine their usefulness and limitations. The observations have been summarised in the Journal of Medical Internet Research (JMIR) article by Woo and Ng [19].

The course curriculum contained a compilation of a series of lectures with various topics in radiation therapy. The first class (2001) consisted of 7 graduate students who were enrolled in a regular Master of Medical Physics program from the University of Malaya, covering both imaging and therapy courses. The topics for the tele-education course were then chosen to supplement the regular program, with special attention paid to hands-on demonstration of software packages, and topics requiring more interactive communication between the two sides.

**Figure 1:**
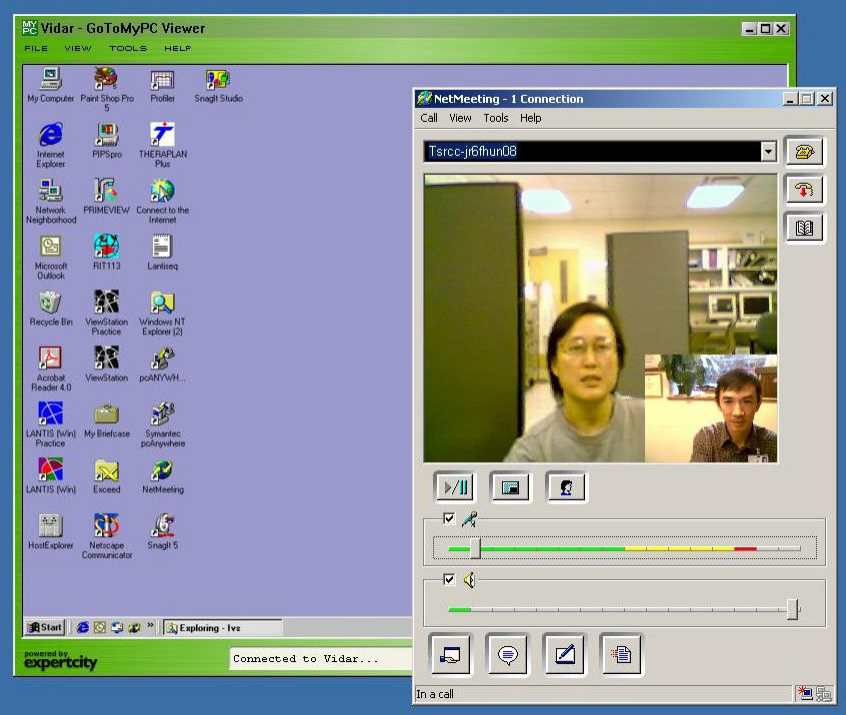
Typical scenario of a lecture. The instructor’s PC screen appears on that of the students’ PC, together with the video picture of both parties using the software program NetMeeting.

**Figure 2:**
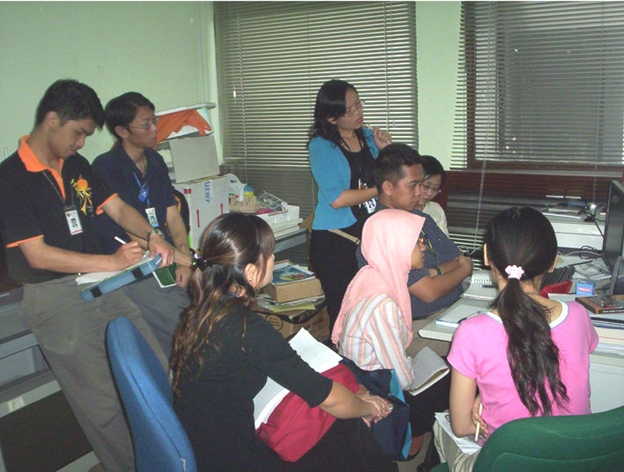
A class of students at the University of Malaya in an actual teleteaching session.

A series of one-hour lectures, including software demonstration, was given during the course of the project. Figure 1 shows a scenario of the lecture, where the instructor’s PC screen appears on that of the students’ PC, together with the video picture of both parties using the program NetMeeting [23]. Figure 2 shows a class of students at the University of Malaya in an actual teleteaching session while Video 1 is a video clip showing the class of students in an actual session. The details of that pilot study, including students' evaluation of the project, are described in the JMIR article [19].

**Video 1:** A video clip showing the class of students in an actual session.

### Project with Wuhan University

This is a new project that has just been launched as a collaboration between the Medical Physics Department at Wuhan University in Wuhan, Hubei, China and the Odette Cancer Centre, at the University of Toronto in Canada.

The Medical Physics Department at Wuhan University is relatively new, with a young faculty whose experience is mainly in the imaging area. In the past, the therapy aspect of the program had been provided by external consultants affiliated with the university. Currently, the radiation therapy programs in China are undergoing rapid advancements, resulting in increasing demand for well-trained medical physicists. There are only three major medical physics programs in China, of which Wuhan is the newest. This particular project conducts classes on a regular basis for about 10 students who are in the second year of a 3-year graduate medical physics program. The initial objective is to assess the level of the students and to identify areas where this mode of distant education can benefit the program, with the possibility of expanding the project into a full course if appropriate and necessary.

In other words, a definable objective is to establish the synchronous mode of distant education to offer a standard radiotherapy physics course on a regular basis, with supplementary help from the local faculty of the university or hospital.

## SOME OBSERVATIONS AND EXPERIENCES GAINED FROM THE PROJECTS

Some important and useful experiences were gained from the first pilot project and have been reported [19]. These observations are reproduced and summarised below:

### Time difference

The time difference between the two locations was 12 hours (13 during daylight-savings time - same for both projects). At first sight, this seemed to pose a significant challenge, and probably would have prevented any process that required dedicated networking facilities available only at universities or hospitals. With the prevalence of high-speed Internet at home, however, this turned out to be quite easily accommodated. Indeed, it was found to be more practical for the instructor to deliver the lecture from home in the evening, although the times had to be reversed for some of the workshops where the software was not available on the instructor’s laptop. In this situation, the instructor demonstrated the software at the office in the morning, while the students held the class at the university in the evening. Once the students were familiar with the software, they were allowed to log onto the instructor’s office computer using a guest account and run the software program independently. The students also managed to download the PowerPoint slides for offline viewing.

### Communication Method

#### Screen-sharing software

For the first pilot project, the screen-sharing system GoToMyPC [21] required a subscription. Compared to most other synchronous service providers, it was found to be the most suitable one for our requirements because of its ease of use, ability to work properly in the presence of firewalls, and relatively low cost.

In the current project with Wuhan University, another software platform, Microsoft's LiveMeeting [24], is used. The main advantage of this platform is that it offers the flexibility of having multiple sites, for both the lecturer and the students. This function is very useful since a projector is not available in the classroom, so the lecture can be viewed at multiple workstations. In the future this can also provide flexibility to link up students at other locations. In addition, the multi-site lecturer feature allows excellent flexibility to recruit experts to participate in a regular weekly course.

Many other platforms for PC-based video-conferencing have undergone a lot of advancements in the last few years and are very well-established. A good summary review is available [10].

#### Audio communication

Effective audio communication was very critical to the success of the lectures. It was found that using the regular telephone network was most reliable, and the cost was relatively low.

However, using a fixed land-based speakerphone was not as straightforward as one would assume. In fact, telephone service was not as easily accessible as Internet service in many classrooms. This posed a significant challenge to our ongoing project at Wuhan. Fortunately, voice-over-IP (VoIP) telephone service using the Internet has become very advanced over the last few years [25], offering a viable solution for our application. The software package Skype [22], which offers free PC-to-PC audio and video connection, was used for the pilot project in University of Malaya as well as the Wuhan project. However, the free service provided by Skype was often unreliable, especially when Internet bandwidth was limited. In these situations, switching to a paid VoIP service improved the quality significantly. Even then, the connection could degrade over the time of the lecture, and the participants would have to disconnect and redial again. The project also tested the use of dedicated video-conferencing connections and fixed phone-line connections, and discovered that sub-optimal audio quality was frequently experienced as well. In conclusion, the audio quality of the lecture is very critical, even more so than video quality, so this aspect of the lectures has to be optimised.

#### Video communication

In the Malaysian pilot study, there was some success with the software NetMeeting, but problems often arose due to firewall issues. However, it is interesting to note that the lectures managed quite well without video communication, although it did serve as a valuable feedback mechanism.

With the Wuhan project, video was transmitted via Skype or LiveMeeting. The experience with LiveMeeting was not satisfactory. However, Skype was often more acceptable especially if the audio communication was handled separately, for example through another PC running VoIP. Again, video linkage was not critical for the lectures, but it added to the quality of the synchronous experience.

#### Firewall

In the beginning, the presence of a firewall proved to be a major hurdle which prevented the use of a lot of common software tools such as MSN messenger [26], Yahoo Messenger [27], QQ [28], etc. On the one hand, the simple combination of GoToMyPC and the telephone proved to be sufficient to complete a series of one-hour lectures, but on the other hand, if a connection could be established without the firewall then the method of communication could be more flexible and powerful. It is recommended that the computer department of the organisation or university be involved to help solve the issue of the firewall.

### Course contents

A standard medical physics curriculum already existed for the Master of Medical Physics program at the University of Malaya, so a series of special topics, such as IMRT (Intensity Modulated Radiation Therapy), was selected for the lectures. A demonstration of a treatment planning system was also carried out. It was found that lectures delivered using Power Point slides were the easiest to conduct, but topics such as ‘Dose Calculation’, which would be most beneficial using the synchronous mode of delivery, require more blackboard-type illustrations and lecturer-student interactions. A whiteboard feature in the software (GoToMyPC and LiveMeeting) allowed this to take place, and turned out to be very useful even though some practice was required to be familiar with the drawing tools.

### Class and Instructor Interest

The students in both projects welcomed the opportunity to interact directly with foreign experts in the field. Moreover, instructors also found the experience interesting and rewarding. The technology did pose a lot of challenges and demanded a lot of patience, especially in the initial trial-and-error stages, but the flexibility and the usability of the process rendered the overall experience very positive.

### Cost

It is encouraging to report that the cost of the remote-education program was kept to a bargain level. This, of course, depended on the availability of fast Internet access. The software program used cost about US$100 per year to install on the instructor’s computer and could be used legally and freely by the students. The other costs such as phone costs, minor hardware items, etc., were insignificant compared to the travel costs that would have been incurred to attend workshops. The major cost that remained was the lecturer’s fees, but since the process offered the lecturer a great deal of flexibility in terms of time and place for the lectures, the lecturer’s cost could also be kept to the minimum. This is a new and exciting process that many people in the field will be interested to participate in. Hopefully this will encourage lecturers to donate their time for an affordable and worthwhile project.

## CONCLUSION

To start a real-time interactive remote education program, careful planning must be undertaken. In a recent case study on using the Internet to teach health informatics [29], various technical difficulties, in particular for synchronous communication, were discussed. Computer and network problems can be very frustrating and minor glitches can discourage both the instructors and students. It is highly recommended that a new program should be carried out with a minimal but reliable set of tools, such as the standard telephone used in our project, and preferably with the support of knowledgeable computer consultants.

In addition, it is crucial to have extensive support from the local department at the students' university or hospital, in terms of IS (Information Systems) expertise as well as organisational assistance, such as a local course organiser or teaching assistant. These are people who are more familiar with the local requirements, as well as protocols, procedures and limitations. When the course is deemed to provide real and tangible value, then the organisational and technical aspects of the synchronous delivery will be much smoother.

In conclusion, the project has proven that real-time interactive remote education of medical physics is a viable concept that is ready to be carried out for selected places and groups. It serves a useful purpose and is a very cost-effective way to promote closer communication and dissemination of knowledge and information within the medical physics community. There are certainly limitations and any new programs must be carefully planned. The increased usage of this method will more speedily eliminate some of the existing limitations.
